# Differential expression of conserved and novel microRNAs during tail regeneration in the lizard *Anolis carolinensis*

**DOI:** 10.1186/s12864-016-2640-3

**Published:** 2016-05-05

**Authors:** Elizabeth D. Hutchins, Walter L. Eckalbar, Justin M. Wolter, Marco Mangone, Kenro Kusumi

**Affiliations:** School of Life Sciences, Arizona State University, Tempe, AZ 85287 USA; Neurogenomics Division, Translational Genomics Research Institute, Phoenix, AZ 85004 USA; Department of Bioengineering and Therapeutic Sciences, Institute for Human Genetics, University of California San Francisco, San Francisco, CA 94143 USA; Virginia G. Piper Center for Personalized Diagnostics, The Biodesign Institute at Arizona State University, Tempe, AZ 85287 USA

**Keywords:** microRNA, Regeneration, Transcriptome, Lizard, Reptile, Gene expression, Tail, Muscle, Brain

## Abstract

**Background:**

Lizards are evolutionarily the most closely related vertebrates to humans that can lose and regrow an entire appendage. Regeneration in lizards involves differential expression of hundreds of genes that regulate wound healing, musculoskeletal development, hormonal response, and embryonic morphogenesis. While microRNAs are able to regulate large groups of genes, their role in lizard regeneration has not been investigated.

**Results:**

MicroRNA sequencing of green anole lizard (*Anolis carolinensis*) regenerating tail and associated tissues revealed 350 putative novel and 196 known microRNA precursors. Eleven microRNAs were differentially expressed between the regenerating tail tip and base during maximum outgrowth (25 days post autotomy), including *miR-133a*, *miR-133b*, and *miR-206*, which have been reported to regulate regeneration and stem cell proliferation in other model systems. Three putative novel differentially expressed microRNAs were identified in the regenerating tail tip.

**Conclusions:**

Differentially expressed microRNAs were identified in the regenerating lizard tail, including known regulators of stem cell proliferation. The identification of 3 putative novel microRNAs suggests that regulatory networks, either conserved in vertebrates and previously uncharacterized or specific to lizards, are involved in regeneration. These findings suggest that differential regulation of microRNAs may play a role in coordinating the timing and expression of hundreds of genes involved in regeneration.

**Electronic supplementary material:**

The online version of this article (doi:10.1186/s12864-016-2640-3) contains supplementary material, which is available to authorized users.

## Background

Among amniotes, while mammals and birds display only limited capacity for regeneration in the adult, lizards retain the ability to regrow their tails, including the formation of multiple tissues such as spinal cord, skeletal muscle, vasculature, cartilage, and skin, throughout their lives [1–8]. Transcriptomic analysis of the green anole lizard, *A. carolinensis*, regenerating tail revealed differential expression of genes involved in wound response, hormonal response, and musculoskeletal development as well as the Wnt and MAPK/FGF pathways [4]. This study and others have demonstrated that the regenerating tail is not a recapitulation of development but a different structure with the same function [4, 7]. While many orthologous genes can be identified between the genomes of the green anole and mammals such as mouse and human [9], a key question about the evolution of regeneration in vertebrates focuses on what genetic changes are responsible for lizards retaining their regenerative capacity and mammals and birds losing this ability.

Changes in the coding or cis-regulatory sequences of multiple individual genes could account for the differential capacity for regeneration within vertebrates. However, given the large number of genes regulating this process, regulators of multiple genes may be involved. MicroRNAs can modulate the expression levels of large numbers of genes, and divergent microRNA regulation could contribute to differences in regeneration between reptilian and mammalian vertebrates. MicroRNAs are highly conserved across metazoa [10] and play critical roles in regulating a variety of biological processes, including proliferation and differentiation of neurons as well as cardiac and skeletal muscle tissue during development [11], hematopoietic and embryonic stem cell differentiation [12, 13], and T-cell development, maturation, differentiation, and activation [14]. MicroRNAs also play a key role in regulating muscle development and repair, which has been extensively studied in mouse and other model systems [15]. The role of microRNA regulation in adult regeneration is an active area of research in vertebrate models.

The expression of microRNAs during development and regeneration has been investigated in amphibians (including the axolotl, the newt, *Xenopus* adult and tadpoles) and in teleosts such as the zebrafish. In the axolotl, microRNAs regulate limb and tail regeneration [16–18]. In the newt, distinct sets of microRNAs, specifically the let-7 family, are expressed during lens and inner ear hair cell regeneration [18, 19]. In zebrafish, microRNAs play an important role in heart, spinal cord, and caudal tail fin regeneration [20–22].

MicroRNAs from whole animal for the green anole lizard have been reported [23], but no studies have been carried out to identify microRNAs in tail regeneration of any lizard species. To investigate the role of microRNAs in lizard regeneration, we performed deep sequencing of RNA smaller than 100 bp. We targeted our analysis on microRNAs from two distinct regenerating tail tissues, the growing tip and base, which yielded differentially expressed transcripts on total RNA transcriptomic analysis [4]. MicroRNA profiles from adult brain and skeletal muscle were assayed to help in annotation of small RNAs. From this sequencing data and subsequent microRNA annotation, we identified differentially expressed microRNAs between the growing tip and base of the regenerating tail that may play important roles in regulating stem cell proliferation and differentiation during regeneration. Furthermore, we predicted the mRNA targets of lizard microRNAs and correlated their expression with mRNA expression identified in a previous study [4]. This study advances our understanding of which post-transcriptional regulators may regulate regenerative capacity in the lizard.

## Results

### Identification of microRNAs in the regenerating lizard tail

**Fig. 1:**
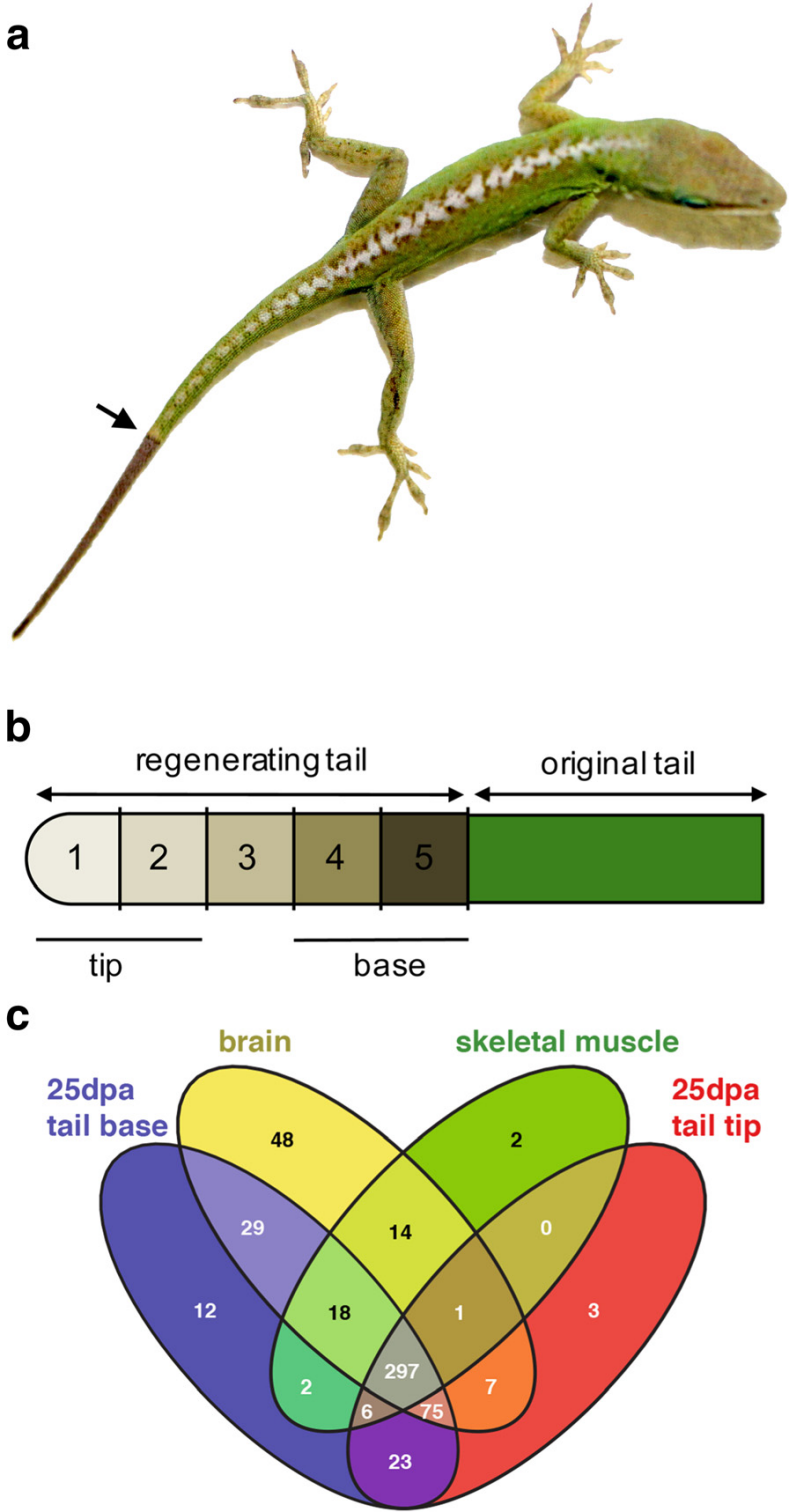
Experimental design of microRNA analysis of lizard tail regeneration. **a**: Image of a green anole lizard with a fully regenerated tail (arrow at break point). **b**: A 25 dpa regenerating tail was divided into three equally sized segments, with the distal regenerating tip and proximal regenerating base collected for microRNA sequencing (sequenced each for the regenerating tail tip and base, n = 3 per pool). For qRT-PCR analysis, five equally sized segments were collected (n = 4). **c**: Venn diagram showing the distribution of microRNAs expressed in the brain, skeletal muscle, and 25 dpa regenerating tail tip and base (minimum count of 1)

During tail regeneration in the green anole lizard, there is rapid outgrowth at 25 days post autotomy (dpa). We collected nine regenerating tails at this 25 dpa stage and dissected and pooled tissue from the tip and base to obtain sufficient RNA for sequencing (*n* = 3 per pool; 3 pools as biological replicates) (Fig. 1a-b; Table 1). These regenerating tail tissues and stages corresponded to our previous RNA-Seq gene expression analysis, permitting comparison of microRNA and mRNA levels [4]. The 326 differentially expressed genes identified in our previous study clustered into two groups characterized by elevated gene expression in the regenerating tail tip or base. Therefore, we sought to identify microRNAs in these tissues that could regulate the regenerative process. To aid in annotating putative novel microRNAs and confirm the presence of previously identified microRNAs in the green anole, we sequenced microRNAs in adult skeletal muscle and brain, which represent component tissues of the regenerating tail (muscle and central nervous system).

**Table 1:** MicroRNA sequencing read summary

Sample	Number of Reads					Number of microRNA precursors	
	Sequenced	Adapter Trimmed	Quality Filtered	Unique	Mapped to Anocar2.0	Known	Putative Novel
ALL TISSUES	58,931,365	51,635,802	48,210,322	1,704,571	642,584	196	350
Regenerating Tail Tip (replicate pool 1)	6,896,312	4,911,787	4,638,573	267,572	80,047		
Regenerating Tail Tip (replicate pool 2)	8,771,826	7,690,607	7,073,991	213,808	67,955		
Regenerating Tail Tip (replicate pool 3)	8,738,345	8,054,177	7,339,012	205,521	84,089		
Regenerating Tail Base (replicate pool 1)	6,905,196	6,084,203	5,763,610	317,605	134,040		
Regenerating Tail Base (replicate pool 2)	9,398,842	8,815,680	8,181,644	245,564	87,691		
Regenerating Tail Base (replicate pool 3)	5,898,914	5,514,428	5,107,890	157,094	62,711		
Adult Skeletal Muscle	3,510,208	2,890,930	2,744,587	124,822	48,387		
Adult Whole Brain	8,811,722	7,673,990	7,361,015	172,585	77,664		

Annotation was carried out using miRDeep2 [24, 25], a tool designed to identify known and putative novel microRNAs from small-RNA sequencing together with the miRBase database of published microRNAs [26, 27]. MiRDeep2 takes into account predicted microRNA secondary structure and uses the expression of 5p and 3p mature sequences in order to assign a score to each novel microRNA precursor. Our miRDeep2 analysis identified a total of 546 precursor microRNA families using a miRDeep2 score of 5 (corresponding to a true positive rate of 94 ± 1 %) for putative novel microRNAs (Additional file 1: Table S1; Fig. 1c). This compares to 282 microRNA anole precursors already identified in miRBase [23]. Of the 546 precursor putative microRNAs that we identified from regenerating tail, brain, and skeletal muscle, 196 of these precursors were also present in miRBase (Additional file 1: Table S1). The remaining 350 putative microRNA precursors were identified by miRDeep2 as potentially novel. Of these, 215 are most likely orthologs of microRNAs found in other systems, displaying either 100 % seed identity or a reciprocal BLAST hit to vertebrate microRNA precursors found in miRBase. This left 135 putative microRNA precursors with no currently known ortholog based on sequence alone (Additional file 1: Table S1) [26, 27]. Analysis of synteny conservation of these microRNA precursors did not identify any clear orthologs in the mouse or human based on genomic location.

### Tissue-specific patterns of microRNA gene expression

Altogether, 12 microRNAs are uniquely expressed in the regenerating tail base compared to only three anole microRNAs identified in the regenerating tail tip (Fig. 1c). Most highly expressed microRNAs in regenerating tissue are expressed in both the tip and the base of the regenerating tail (Table 2). While most microRNAs are shared amongst tissues, the brain displayed the largest number of unique microRNAs (Fig. 1c). 489 microRNAs were expressed in brain, 340 are expressed in skeletal muscle, and 473 were expressed in regenerating tail tissue. Highly expressed microRNAs in the brain include a number of regulators of neuronal development and differentiation such as *miR-124a*, *miR-124b*, *miR-9*, and *miR-26* (Table 2) [28–33]. *miR-124a*, *miR-9*, and *miR-181a* specifically are some of the most abundant microRNAs expressed in the vertebrate central nervous system [34–36]. Highly expressed microRNAs in the skeletal muscle include the muscle specific microRNAs, or myomiRs, *miR-1* and *miR-133a* [37, 38], along with *miR-26*, *miR-125b*, and *miR-27* all of which are involved in myogenesis and skeletal muscle repair (Table 2) [39–42]. Having identified the tissue specificity of the identified microRNAs, we focused on differential expression within the regenerating tail.

**Table 2:** Highly expressed microRNAs in brain, skeletal muscle, and regenerating tail tip and base (DESeq normalized counts)

microRNA precursor	brain	microRNA precursor	muscle	microRNA precursor	regen. tail tip	microRNA precursor	regen. tail base
aca-mir-124b	96,714	aca-mir-1a-1	144,296	aca-mir-21	212,122	aca-mir-21	187,018
aca-mir-125b-1	75,541	aca-mir-1a-2	144,242	aca-mir-10b	78,808	aca-mir-199b	60,669
aca-mir-125b-2	73,617	aca-mir-133a-1	55,682	aca-mir-27b	67,317	aca-mir-27b	56,981
aca-mir-99b	64,863	aca-mir-133a-2	55,682	aca-mir-199b	65,229	aca-mir-199a-2	55,663
aca-mir-26-2	43,364	aca-mir-26-2	43,039	aca-mir-199a-2	29,690	aca-mir-199a-1	55,631
aca-mir-26-1	43,234	aca-mir-26-1	42,941	aca-mir-199a-1	29,657	aca-mir-10b	55,446
aca-mir-125a	41,711	aca-mir-21	33,124	aca-mir-203	29,477	aca-mir-99b	34,538
aca-mir-124a-2	39,123	aca-mir-99b	28,191	aca-mir-26-2	28,893	aca-mir-26-2	32,110
aca-mir-124a-1	39,122	aca-mir-124b	26,041	aca-mir-26-1	28,813	aca-mir-26-1	32,022
aca-mir-124a-3	39,122	aca-mir-125b-1	23,844	aca-mir-99b	20,212	aca-mir-203	25,853
aca-mir-100	30,873	aca-mir-27b	23,384	aca-mir-10a	18,919	aca-let-7a	17,446
aca-mir-9-3	22,674	aca-mir-125b-2	23,191	aca-mir-205a	16,783	aca-mir-10a	16,705
aca-mir-9-1	22,665	aca-mir-143	16,657	aca-let-7f-1	16,737	aca-let-7f-1	16,311
aca-mir-9-2	22,665	aca-mir-99a	12,634	aca-let-7a	16,118	aca-mir-1a-1	16,175
aca-let-7c-1	21,340	aca-mir-125a	12,331	aca-let-7f-2	15,753	aca-mir-1a-2	16,150
aca-let-7c-2	21,340	aca-mir-124a-2	11,107	aca-mir-181a-3	14,394	aca-let-7f-2	15,625
aca-mir-99a	20,749	aca-mir-124a-1	11,107	aca-mir-181a-2	14,348	aca-mir-140	14,167
aca-let-7a	19,748	aca-mir-124a-3	11,107	aca-mir-181a-1	14,347	aca-mir-148a	11,433
aca-mir-27b	16,237	aca-mir-100	10,673	aca-let-7e	10,906	aca-let-7e	11,273
aca-mir-181a-3	12,498	aca-mir-451	10,081	aca-mir-148a	10,513	aca-let-7c-1	10,238

### Differential expression analysis of regenerating tail microRNAs and coordinated expression with mRNAs

**Fig. 2:**
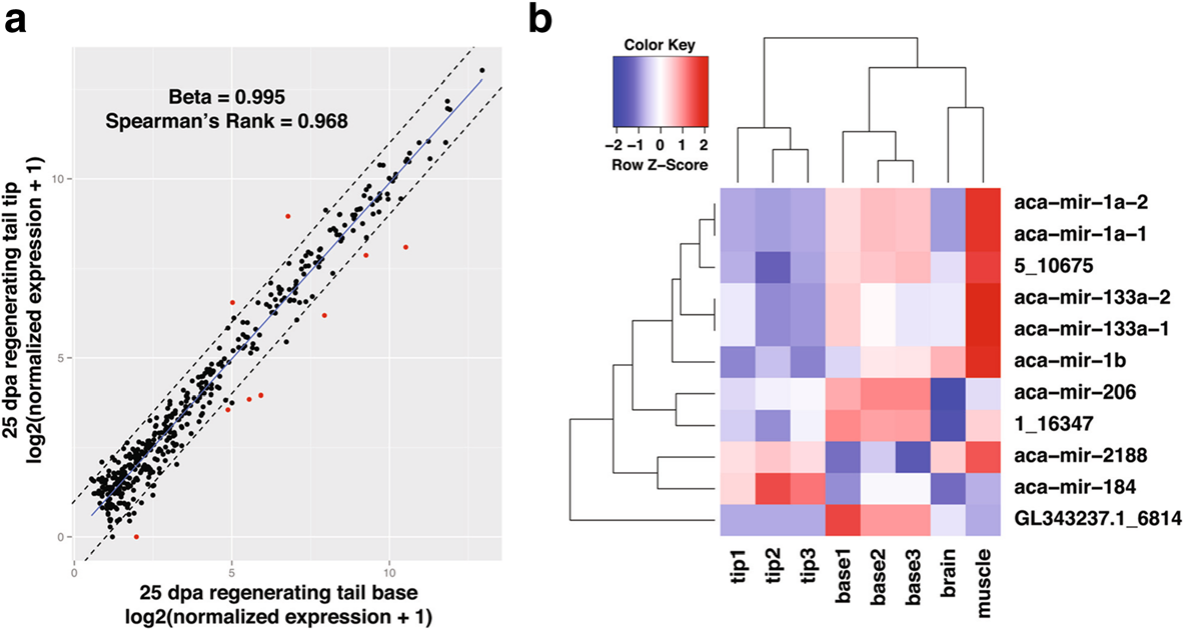
Differential expression of microRNAs in the 25 dpa regenerating lizard tail. **a:** Regression of normalized microRNA expression in the regenerating tail tip and base (Beta-value = 0.995, Spearman’s Rank = 0.968). Each point on the graph represents a microRNA. Dashed lines represent the cutoff for two-fold change. Differentially expressed microRNAs, i.e., displayed significant expression differences as determined by DESeq (adjusted *p* < 0.05) are represented in red. **b:** Heatmap of differentially expressed microRNAs. Expression in each replicate is shown. MicroRNAs were clustered by Jensen-Shannon divergence of DESeq variance stabilization transformed expression data

Small-RNA sequencing of the 25 dpa regenerating lizard tail tip and base identified the expression of 546 microRNAs (Additional file 2: Table S2). In general, most of the microRNAs were highly correlated between these two tissues, with only 11 differentially expressed microRNAs (Fig. 2a; Additional file 3: Table S3; adjusted *p* < 0.05). The impact of differential expression of 11 microRNAs is of course amplified by a larger number of predicted target genes (Table 3; Additional file 4: Table S4) [43].

**Table 3:** Predicted mRNA targets of upregulated, differentially expressed microRNAs (orthologous and putative novel)

Regenerating tissue source	Upregulated, differentially expressed microRNA	Predicted gene targets
regenerating tail tip	aca-miR-184	*adprhl2*, *anpep*, *ar*, *b3gnt1*, *bai2*, *ccar1*, *ccdc50*, *cdkn2c*, *cox4i2*, *depdc5*, *fam160a2*, *gpi*, *h6pd*, *kars*, *limk1*, *me1*, *ncan*, *nek6*, *oxnad1*, *pcsk4*, *pdia3*, *pdpk1*, *phlda1*, *ring1*, *rtn2*, *slc30a2*, *slc43a1*, *suclg1*, *sugp2*, *tgfb1*, *tmem214*, *usp21*, *xxylt1*, G.11044, G.14682, G.15668, G.19327, G.19921, G.20484, G.21669, G.21917, G.21923, G.22240, G.22349, G.22365, G.22632
regenerating tail tip	aca-miR-2188	*aaas*, *b3gnt7*, *crtac1*, *grm7*, *hsf1*, *hsp90b1*, *itpkb*, *itsn1*, *mta1*, *mylk2*, *nmnat3*, *pou2f2*, *tpd52*, *zbtb45*, G.1698, G.6382
regenerating tail base	1_16347 (putative novel)	*abcf3*, *adhfe1*, *amn1*, *c11orf35*, *cep76*, *chrna4*, *ddit4*, *dmtn*, *dpy30*, *dpysl3*, *fam57a*, *ikzf3*, *jarid2*, *lrrc4b*, *moxd1*, *mtpap*, *nmnat2*, *pfdn4*, *rps6kl1*, *scarf2*, *smpd2*, *sox13*, *sws2*, *tlk2*, *tpt1*, *tpx2*, *trmt1*, G.11229, G.11992, G.14528, G.16037, G.19728, G.19926, G.4056
regenerating tail base	5_10675 (putative novel)	*adam33*, *ap1b1*, *arhgef33*, *ccdc104*, *efcab4a*, *fermt2*, *ggt1*, *klhl38*, *ncoa4*, *nkd1*, *pipox*, *plxna4*, *ppfia4*, *psmc6*, *psmf1*, *rgs18*, *sall1*, *sdf2l1*, *traf3ip3*, *trim65*, *txlna*, *zfyve1*, G.11978, G.20962, G.21441, G.4400
regenerating tail base	aca-miR-1b	*efhd1*, *irak4*, *sema4c*, *slain2*, *snai2*, *tktl1*, G.22875, G.9382
regenerating tail base	aca-miR-206	*ankrd17*, *c5orf30*, *cd44*, *cep192*, *chrac1*, *gbe1*, *notch3*, *poldip3*, G.14293, G.4173, G.9382
regenerating tail base	GL343237.1_6814 (putative novel)	*ddb2*, *elmsan1*, *irf7*, *kank4*, *kifap3*, *klhdc3*, *ldb2*, *map1lc3b*, *nfia*, *orc4*, *ppp1r9b*, *ptprh*, *secisbp2*, *swap70*, *vash2*, *znf385c*, G.12700, G.2381, G.3078, G.4859
regenerating tail base	aca-miR-1a-1; aca-miR-1a-2	*ankrd17*, *efhd1*, *gbe1*, *ikbkap*, *irak4*, *pdgfa*, *sema4c*, *slain2*, *snai2*, *tktl1*, G.14293, G.9382
regenerating tail base	aca-miR-133a-1; aca-miR-133b	*abcf3*, *adhfe1*, *amn1*, *arhgdia*, *c10orf12*, *c11orf35*, *cacna1b*, *cep76*, *cfdp1*, *chrna4*, *col1a1*, *creld1*, *ddit4*, *dmtn*, *dpy30*, *dpysl3*, *fam57a*, *gria1*, *gtpbp1*, *ikzf3*, *lrrc4b*, *moxd1*, *mtpap*, *nmnat2*, *pfdn4*, *ppapdc2*, *rps6kl1*, *scarf2*, *smpd2*, *sox13*, *tm2d3*, *tpt1*, *tpx2*, *trmt1*, *vcp*, G.10949, G.11229, G.11992, G.14528, G.16037, G.19284, G.19728, G.19926, G.3656, G.4056, G.5104

The differentially expressed microRNAs could be clustered into four groups, where many microRNAs upregulated in the base share high levels of expression with skeletal muscle (Fig. 2b). Nine of these microRNAs have elevated expression in the tail base, including *miR-1*, *miR-133a*, *miR-133b*, and *miR-206*, which have been shown to play key roles in regulating skeletal muscle differentiation and function [37, 44–48]. In zebrafish, the miR-133 precursor family regulates regeneration in the tail fin [21], the heart [49], and spinal cord [22]. In mice, *miR-1* and *miR-206* regulate satellite cell proliferation via repression of *Pax7* translation, thereby promoting myotube formation [48, 50]. *miR-184*, which is differentially expressed in the regenerating tail tip, regulates proliferation and differentiation of neural stem cells [51]. Of the 11 differentially expressed microRNAs, three were putatively novel.

**Fig. 3:**
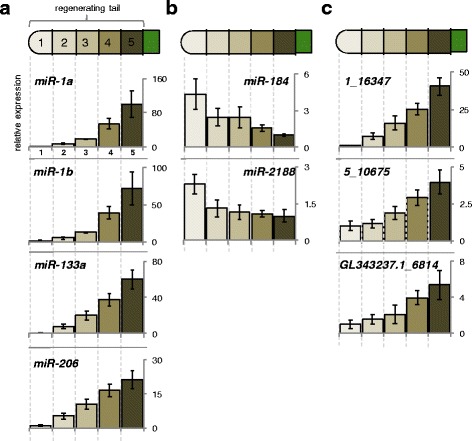
miRNA expression across regenerating tail sections by qRT-PCR. Total RNA was extracted from regenerated tail sections from four biological replicates. cDNA was synthesized using primers specific to the mature miRNA. Three qRT-PCR replicates were performed for each gene in each tail section (600 total reactions). Expression is normalized to *ef1a*, and quantified using the ∆∆Ct method. **a** Highly conserved miRNAs with expression decreasing from the base to the tip. **b** Highly conserved miRNAs with expression increasing from the base to the tip. **c** Expression of putative novel miRNAs across regenerating tail sections

In order to validate and study differential expression of miRNAs in different portions of the regenerating tail we have followed expression levels in 9 miRNAs identified by our sequencing efforts (Fig. 3). We sectioned four regenerated tails into five equal segments, extracted total RNA from each segment, and followed miRNA level changes between these segments using qRT-PCR (Fig. 3). Each miRNA was assayed in triplicate for each tail section, totaling 600 qRT-PCR reactions. As shown in Fig. 3*miR-1a*, *miR-1b*, *miR-133a* and *miR-206* show increased expression in the proximal portion of the regenerating tail, while *miR-184* and *miR-2188* display an opposite pattern. Importantly, we were able to also detect these positional changes in a small subset of putative novel miRNAs (*5_10675* and *GL343237.1_6814*) and putative novel *Anolis*-specific *miR-133b* ortholog (*1_16347)*, detected by our sequencing results (Fig. 3). These three putative novel miRNAs possess hairpin structures indicative of miRNAs (Additional file 8: Figure S1, Additional file 9: Figure S2 and Additional file 10: Figure S3). Additionally, the sequencing reads preferentially stack onto one arm of the hairpin, and have 1–2 nucleotide overhangs, which is characteristic of pre-miRNA processing by Dicer [52] (Additional file 8: Figure S1, Additional file 9: Figure S2 and Additional file 10: Figure S3). Of note, while we detected expression changes in the regenerating tail for *5_10675* by small RNA-Seq and qRT-PCR, this putative novel miRNA maps to multiple regions of the *Anolis* genome, making it difficult to determine the exact genomic origin of the transcript. Taken together, this data validates our miRNA sequencing efforts, and importantly shows differential localization patterns of several miRNAs in the regenerating tail, suggesting that miRNAs may play a functional role in this process.

Predicted targets of these putative novel microRNAs are listed in Table 3. A number of genes predicted to be targeted by the three putative novel microRNAs are involved in mitosis and cell cycle control, including antagonist of mitotic exit network 1 homolog (*amn1*), centrosomal protein 76 kDa (*cep76*), jumonji, AT rich interactive domain 2 (*jarid2*), leucine rich repeat containing 4B (*lrrc4b*), origin recognition complex, subunit 4 (*orc4*), protein phosphatase 1, regulatory subunit 9B (*ppp1r9b*), proteasome macropain 26S subunit, ATPase, 6 (*psmc6*), proteasome macropain inhibitor subunit 1 (*psmf1*), tousled-like kinase 2 (*tlk2*), tumor protein translationally-controlled 1 (*tpt1*), and the microtubule-associated gene *tpx2.* In addition, a number of genes involved in neurogenesis or synapse formation were targets, including cholinergic receptor, nicotinic, alpha 4 (*chrna4*), dihydropyrimidinase-like 3 (*dpysl3*), plexin A4 (*plxna4*), sphingomyelin phosphodiesterase 2 neutral membrane (*smpd2*), and EF-hand domain family member D1 (*sws2/efhd1*). Finally, Wnt pathway members fermitin family member 2 (*fermt2*)*,* naked cuticle homolog 1 (*nkd1*), and spalt-like transcription factor 1 (*sall1*) were among the predicted targets. Given the cell proliferation and tissue formation taking place within the regenerating tail base, these putative novel microRNAs may play a key role in regulating the regenerative process.

**Fig. 4:**
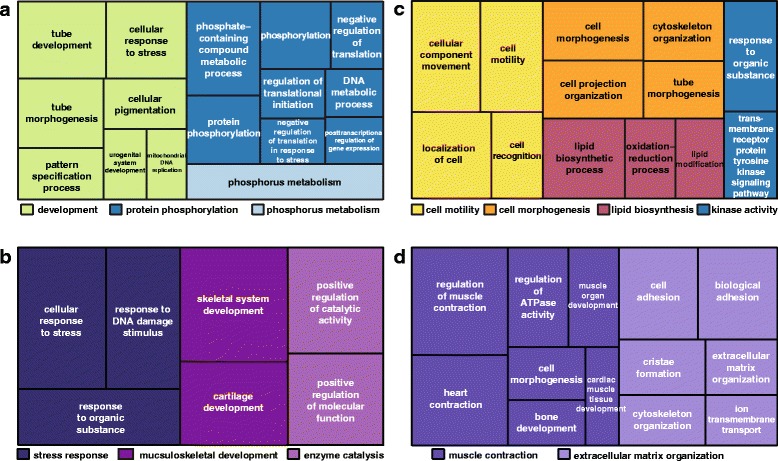
microRNAs and their co-expressed mRNA targets in the 25 dpa regenerating lizard tail. **a**–**b**: A treemap overview of significant (*p* < 0.05) Gene Ontology Biological Processes for downregulated microRNAs and their upregulated mRNA targets in the 25 dpa regenerating tail tip (**a**) and regenerating tail base (**b**). **c**–**d**: A treemap overview of significant (*p* < 0.05) Gene Ontology Biological Processes for upregulated microRNAs and their upregulated mRNA targets in the 25 dpa regenerating tail tip (**c**) and regenerating tail base (**d**). The relative sizes of the treemap boxes are based on the |log10(*p*-value)| of the respective GO term. Related terms are visualized with the same color, with the representative category for each color group denoted in the legend

We have previously shown that there are at least 326 differentially expressed genes in the regenerating lizard tail, including genes in the Wnt and FGF/MAPK pathways as well as those involved in wound repair, hormonal regulation, and musculoskeletal development [4]. We identified microRNA/target mRNA pairs that both have at least 2-fold change in expression between the regenerating tail tip and base (Additional file 5: Table S5) and performed DAVID analysis of Gene Ontology Biological Processes on the targeted mRNA transcripts to identify significant functional terms (*p* < 0.05; Additional file 6: Table S6) [53, 54]. Of particular interest are coordinated profiles of expression where the microRNA changes reinforce the mRNA gene expression, i.e., microRNAs levels are decreased where the expression of their mRNA targets are increased, as these could represent post-transcriptional microRNA repression (Fig. 4a-b). Additionally, the group of highly expressed mRNA genes whose regulatory microRNAs are also upregulated in the corresponding tissue are of interest as they could represent translational microRNA repression (Fig. 4c-d).

In the regenerating tail tip, genes involved in phosphorus metabolism, phosphorylation, development of tubular structures, cell motility, cell morphogenesis, lipid biosynthesis, and kinase activity are highly expressed. This would be expected in the regenerating tail tip, where organization of structures with epithelial cell organization such as the vasculature, ependymal, and cartilage tube would require active signal transduction via phosphorylation (Fig. 4a, c). MicroRNAs that reinforce this pattern of expression are all putatively novel. In the regenerating tail base, genes involved in musculoskeletal development, enzyme catalysis, response to organic substances, muscle contraction, and extracellular matrix organization display increased expression, as might be expected in differentiating skeletal muscle and cartilage present in that tissue (Fig. 4b, d). MicroRNAs that reinforce this pattern of expression included many putative novel microRNAs, including two of the differentially expressed putative novel microRNAs (*5_10675* and *1_16347*), as well as *let-7b*, which is regulates neural stem cell proliferation and is additionally expressed during lens regeneration in the newt [18, 19, 55].

## Discussion

This study describes the first microRNA transcriptome analysis of regeneration in the green anole. We identified 546 microRNA precursors from regenerating tail and adult brain and skeletal muscle, with 411 microRNA precursors orthologous to families in other vertebrate species. In addition, we have validated the presence of a subset of differentially expressed miRNAs detected by our sequencing efforts, including three putative novel miRNAs. Given previous analysis finding a distributed pattern of cell proliferation throughout the regenerating green anole tail [4], we did not expect that the tail tip would be enriched for stem cell proliferation or reveal a gradient of differentiation. The cellular organization at the tail tip differs from the base in being enriched for forming vasculature, growing ependyma, and coalescing cartilage tube. In addition, we have validated the presence of a subset of miRNAs detected by our sequencing efforts, including three putative novel miRNAs. Differentially expressed mRNAs and microRNAs both reflect this enrichment for those tissues in the tail tip. Conversely, the regenerating tail base is enriched for differentiating skeletal muscle groups, and this pattern was observed in mRNA and microRNA expression. The finding that three putative novel microRNAs are differentially expressed in the regenerating tail is intriguing. However, these putative novel microRNAs likely have homologues in other vertebrates, but the lack of reptilian genomes and microRNAs sequenced to date limits our ability to clearly identify orthology.

Though microRNA target prediction is a useful tool, prediction algorithms often have varying target lists, and their false positive and false negative rates are difficult to assess [56, 57]. While comparing microRNA expression with the expression of its mRNA target helps resolve and identify microRNA/mRNA target pairs for further analysis, it would be beneficial to further verify these genes for downstream analyses. Since microRNAs are an example of post-transcriptional regulation, the addition of proteomic data would provide a unique insight into verification of microRNA targets. Specifically, proteomic data would help assess whether certain microRNAs act at a post-transcriptional or translational level; aiding in resolution of identifying microRNAs that inhibit translation versus false positives in cases where both a microRNA and its targeted mRNA transcript are upregulated.

Given that most microRNA precursors expressed in lizard tail regeneration have orthologs in other vertebrates, comparison with microRNAs identified in other regenerative models could be instructive. For example, the small RNA *miR-133* is downregulated during heart regeneration and in the tip of the regenerating tail in zebrafish [49]. In the anole, we identified high levels of *miR-133a* in the regenerating tail base compared to the tail tip. The small RNA *miR-184*, which is differentially expressed in the tip of anole regenerating tail, has also been identified in zebrafish tail fin regeneration [20]. In addition to regulating neural stem cell proliferation and differentiation, *miR-184* targets the RNA-induced silencing complex (RISC) member argonaute2 [51, 58, 59]. During newt lens regeneration, *miR-1* and *miR-206* regulate cell proliferation [19]. Orthologs of these two microRNAs are both differentially expressed in the regenerating anole tail base. While previous studies did not identify novel microRNA precursors specific to regeneration, we identified 3 previously unknown differentially expressed microRNAs in the regenerating tail base. This may reflect the ability of RNA-Seq to identify novel sequences, while microarray analysis is limited by probe sets included in the arrays. Comparative analysis of the role of microRNAs in vertebrate regeneration would be advanced by further deep sequencing of small RNA populations in other model systems.

## Conclusions

Given that microRNAs are able to regulate a large number of genes, it is possible that microRNA regulation during the regenerative process can contribute to differences in regenerative capacity among vertebrates. Divergence in vertebrate microRNA regulation could arise by a number of possible models including, i.) the deletion or loss of microRNAs regulating regeneration within the mammalian lineage, ii.) the change in downstream transcripts targeted by microRNAs in the mammalian lineage, and iii.) the emergence of novel reptile-specific microRNAs that promote regeneration. The latter model appears less parsimonious given the conservation of regeneration across vertebrates, including teleosts, amphibians, and amniotes (in lizards). In addition to microRNA-based regulation, genomic changes may of course affect coding genes and non-coding regulatory sites such as enhancers, silencers, and insulators. Further analysis in the lizard and comparison with other regenerative models will allow us to further distinguish between these possibilities.

## Methods

### Animal care and tissue collection

All animals were collected and maintained according to Institutional Animal Care and Use Committee guidelines at Arizona State University, which granted ethics approval for this study (Protocol Number 12-1247R). Adult *A. carolinensis* lizards were purchased from Charles D. Sullivan, Inc. (Nashville, TN) or Marcus Cantos Reptiles (Fort Myers, FL) and housed as described previously [4, 60]. Autotomy was induced by firmly holding a point on the tail 5 cm from the base, while the lizard was otherwise allowed to move on a flat surface. Regenerated tails were then collected 25 days post autotomy (dpa). For microRNA isolation for small RNA-Seq, 25 dpa regenerating tails were cut into three sections each, representing the base, middle, and tip of the regenerating tail. Three tip and base sections were respectively pooled, leading to three replicates each containing three pooled tail samples for each tip and base tissue sample. For microRNA isolation for qRT-PCR, four 25 dpa regenerating tails were sectioned into five equal segments as in Hutchins et al. [4].

### microRNA sequencing and annotation

Small RNAs were extracted from adult lizard tissues, including 25 dpa regenerating tail base (*n* = 3) and tip (*n* = 3), brain (*n* = 1), and skeletal muscle (*n* = 1), following the miRVana kit protocol (Ambion). Small RNAs were then barcoded for multiplexed sequencing on two Illumina GAIIx lanes, generating single end 40 base pair reads, and raw sequencing reads from the resulting small RNA libraries were demultiplexed through services provided by LC Sciences. Using the FASTX-Toolkit (http://hannonlab.cshl.edu/fastx_toolkit/), the adapters used for sequencing (TGGAATTCTCGGGTGCCAAGG) were trimmed from the demultiplexed reads while keeping only reads 18 bp or greater, and trimmed reads were quality filtered by removing all sequencing reads with less than 80 % of the bases with at least a Q20 Illumina quality score. The resulting adapter trimmed and quality filtered reads for each for the samples were then mapped to the AnoCar2.0 repeat masked genome available from Ensembl (Ensembl Build 67) [61] using the miRDeep2 package [24, 25] mapper.pl script with the following options: d, e, h, i, j, m. This generated a collapsed set of non-redundant reads while retaining read counts along with the genomic location of the mapped reads. miRDeep2 was then used to annotate putative novel microRNAs in *A. carolinensis*, as well as validate predicted microRNAs from miRBase. Specifically, (1) mapped reads generated by the mapper.pl script, (2) miRBase predicted microRNAs for *A. carolinensis* [26, 27], and (3) the miRBase microRNA sequence datasets for human, mouse, chicken, frog, and zebrafish were all passed through the miRDeep2.pl script [24, 25]. Putative novel microRNA genes predicted by miRDeep2 are assigned a score based on 5p and 3p read support and secondary structures consistent with the biogenesis of microRNAs. Putative novel microRNAs predicted by miRDeep2 were retained for further analysis if they had a miRDeep2 score of 5 or above, corresponding to an estimated false discovery rate of 6 %.

### Statistical analysis of microRNA expression

To determine microRNA expression levels, the set of collapsed, non-redundant reads from the mapper.pl mirDeep2 script were first aligned to the miRBase microRNAs and putative novel microRNAs predicted by miRDeep2 using the quantifier.pl script as part of the miRDeep2 package. This step produced a raw counts file that was then used as input into the DESeq R/Bioconductor package for further statistical analysis [62, 63]. Differential expression tests in DESeq (adjusted *p* < 0.05) were conducted only for microRNA genes with at least 10 reads of support in each of the samples being tested, using the following parameters: fitType = “local” and sharingMode = “fit-only”.

### Quantitative RT-PCR validation of microRNA differentially expressed in the regenerating lizard tail

Four 25 dpa regenerating tails were sectioned into five equal segments, and total RNA from each segment was extracted using the total RNA protocol for the miRVana kit (Ambion), as in Hutchins et al. [4]. cDNA for EF1A was synthesized using a poly-dT primer and SuperScript III (Thermo-Fisher), and was used for normalization. Taqman miRNA primers (Thermo-Fisher) were used to generate cDNA for each mature miRNA with single base resolution, using Taqman miRNA primers (Thermo-Fisher; Additional file 7: Table S7). The first strand primers were pooled, and 100 ng of RNA was used to generate cDNA with SuperScript III. The qRT-PCR for EF1A was performed using SYBR Select Master Mix (Life Technologies) and custom primers (F: CCGTCGTTCTGGTAAGAAACTGG, R: TTAGCCTTCTGCGCCTTCTGG). The qRT-PCR for mature miRNAs was performed using Taqman Fast Advanced Master Mix (Applied Biosystems). Both the miRNA and EF1A qRT-PCRs were performed in 384 well plates on a QuantStudio Dx (Applied Biosystems). Each miRNA was assayed in triplicate for each tail section, totaling 600 qRT-PCR reactions. Relative expression levels were quantified by the ∆∆Ct method.

### microRNA target prediction

The mRNA targets of the known miRBase and putative novel microRNAs were predicted using RNAhybrid and miRanda against 3′ UTR sequences extracted from the ASU_Acar_v2.2.0 gene annotation [60, 64–69]. The RNAhybrid prediction first calibrates the location and scale parameters of the extreme value distribution for each microRNA by using the RNAcalibrate tool against the 3′ UTR sequences in order to improve the *p*-value calculations for each target prediction for each specific microRNA. These calibrated parameters were then used as input for the d-option for the final RNAhybrid prediction step. Additionally, the minimum free energy parameter was set to -20 kcal/mol with a *p*-value ≤ 0.01. The set of miRanda microRNA target predictions was generated by setting the minimum free energy to -20 kcal/mol and requiring no mismatch in the seed region. Only overlapping microRNA target predictions from both RNAhybrid and miRanda were retained. Additionally, microRNA targets were filtered for transcripts that were the target of two or more microRNAs.

### Comparison of microRNA expression and mRNA target expression

Expression of microRNAs in the regenerating tail was compared to the expression of their mRNA targets, with a cut-off of 2-fold change between the tip and base of the regenerating tail. DESeq was used to determine the expression levels of the known and putative novel microRNAs as outlined above, while corresponding transcript expression levels were determined previously [4]. Transcript-microRNA interactions were then filtered for co-expression of both the microRNA and mRNA in either the tip or base of the regenerating tail. All one or greater DESeq normalized values for expression of microRNAs were retained. Similarly, transcripts were required to have at least a Cufflinks estimated FPKM of 1 or greater in at least one section of the regenerating tail to be retained for further analysis. *P*-values for Gene Ontology (GO) analysis of targeted mRNA transcripts were generated using the Database for Annotation, Visualization, and Integrated Discovery (DAVID) functional analysis tool [52, 53]. Significant GO terms (*p* < 0.05) were mapped with the REViGO online tool (http://revigo.irb.hr), which removes redundant GO terms and visualizes the semantic similarity of remaining terms [70].

## Ethics

Ethics approval for this study was granted by the Institutional Animal Care and Use Committee at Arizona State University (Protocol Number 12-1247R).

## Consent to publish

Not applicable.

## Availability of data and materials

All microRNA raw sequencing data is available from the NCBI Short Read Archive/NIH Bioproject accession number PRJNA278692.

## Additional files

Additional file 1: Table S1. Putative novel and known microRNA precursors in the green anole lizard, *Anolis carolinensis,* predicted by miRDeep2. (XLSX 127 kb) Additional file 2: Table S2. MicroRNA expression in green anole tail regeneration: counts from miRDeep2 and normalized DESeq values. (XLSX 120 kb) Additional file 3: Table S3. DESeq differential expression testing of microRNA between the regenerating green anole tail tip and base. (XLSX 85 kb) Additional file 4: Table S4. List of microRNA targets predicted by both RNAhybrid and miRanda. (XLSX 2990 kb) Additional file 5: Table S5. Expression summary of down- or upregulated microRNAs and their mRNA targets. (XLSX 218 kb) Additional file 6: Table S6. DAVID Gene Ontology Biological Processes analysis results for downregulated microRNA/upregulated mRNA target pairs. (XLSX 53 kb) Additional file 7: Table S7. Primers used for quantitative RT-PCR validation of microRNA differentially expressed in the regenerating lizard tail. (XLSX 43 kb) Additional file 8: Figure S1. Folding structure and read alignment for putative novel, Anolis-specific miR-133b ortholog 1_16347. (PDF 204 kb) Additional file 9: Figure S2. Folding structure and read alignment for putative novel miRNA 5_10675.(PDF 202 kb) Additional file 10: Figure S3. Folding structure and read alignment for putative novel miRNA miR GL343237.1_6814. (PDF 218 kb)

## Abbreviations

DAVID: database for annotation, visualization, and integrated discovery
dpa: days post autotomy
GO: gene ontology
qRT-PCR: quantitative reverse transcriptase PCR
RISC: RNA-induced silencing complex (RISC)
UTR: untranslated region
