# Supplementary material for: Differential expression of conserved and novel microRNAs during tail regeneration in the lizard Anolis carolinensis
Source: BMC Genomics. 2016 May 5;17:339. doi: 10.1186/s12864-016-2640-3 (PMC4858913; doi:10.1186/s12864-016-2640-3)
Supplement: Additional file 8: Figure S1. — Folding structure and read alignment for putative novel, Anolis-specific miR-133b ortholog 1_16347. (PDF 204 kb) [file 12864_2016_2640_MOESM8_ESM.pdf]

miRBase precursor : 1_16347
Total read count : 2062
1_16347 read count : 1786
remaining reads : 276

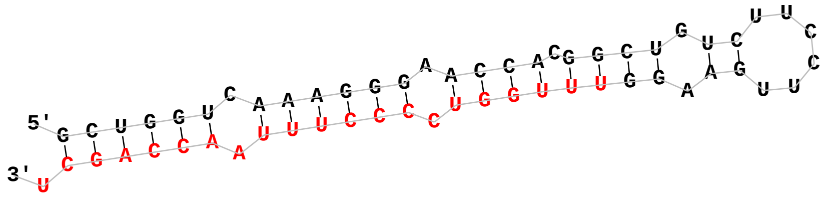

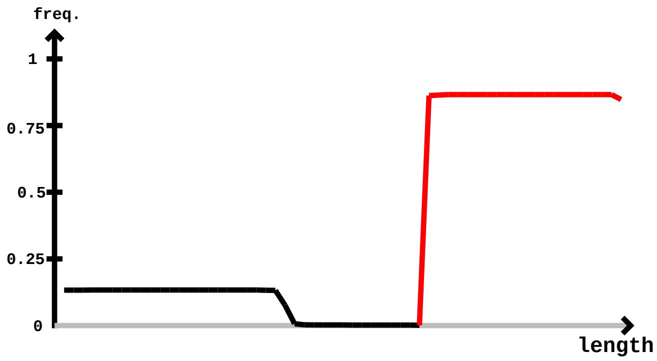


```
                                                        1_16347
5'- gcuggucaaagggaaccacggcugucuuccuugaagguuugguccccuuuaaccagcu   -3'   exp
    ((((((.((((((.((((.((((.((......)).)))))))).)))))).)))))).   reads   mm   sample
    gcuggucaaagggaaccacggcu...................................   1       0    MUS
    .....................................uuugguccccuuuaaccagcu   38      0    MUS
    ......................................uugguccccuuuaaccagcu   1       0    MUS

    gcuggucaaagggaaccacgg.....................................   1       0    TP3
    gcuggucaaagggaaccacggc....................................   14      0    TP3
    gcuggucaaagggaaccacggcu...................................   7       0    TP3
    gcuggucaaagggaaccacggcuU..................................   1       1    TP3
    .....................................uuugguccccuuuaaccagc.   1       0    TP3
    .....................................uuugguccccuuuaaccagcu   86      0    TP3

    gcuggucaaagggaaccacggc....................................   18      0    BS3
    gcuggucaaagggaaccacggcA...................................   1       1    BS3
    gcuggucaaagggaaccacggcu...................................   40      0    BS3
    gcuggucaaagggaaccacggcuU..................................   2       1    BS3
    .....................................uuugguccccuuuaaccagc.   4       0    BS3
    .....................................uuugguccccCuuaaccagcu   1       1    BS3
    .....................................uuuAguccccuuuaaccagcu   1       1    BS3
    .....................................uuugguccccuuuaaccagcuG  1       1    BS3
    .....................................uCugguccccuuuaaccagcu   1       1    BS3
    .....................................uuugguccccuuuaaccagcu   443     0    BS3
    .....................................uuugguccccuuuaaccagcA   2       1    BS3
    ......................................uugguccccuuuaaccagcu   1       0    BS3

    gcuggucaaagggaaccacggc....................................   25      0    BS2
    gcuggucaaagggaaccacggcA...................................   1       1    BS2
    gcuggucaaagggaaccacggcu...................................   35      0    BS2
    gcuggucaaagggaaccacggcuU..................................   2       1    BS2
    .....................................uuugguccccuuuaaccagc.   7       0    BS2
    .....................................uuugguccUcuuuaaccagcu   1       1    BS2
    .....................................uuugguccAcuuuaaccagcu   1       1    BS2
    .....................................uuugguccccuuuaaccagcu   511     0    BS2

    gcuggucaaagggaaccacggc....................................   1       0    BRN
    gcuggucaaagggaaccacggcu...................................   1       0    BRN
```

gcuggucaaagggaaccacggcugucuuccuugaagguuugguccccuuuaaccagcu			
.....................................uuugguccccuuuaaccagcu	23	0	BRN
gcuggucaaagggaGcca..	1	1	TP1
gcuggucaaagggaaccacggc....................................	11	0	TP1
gcuggucaaagggaaccacggU....................................	1	1	TP1
gcuggucaaagggaaccacggcu...................................	14	0	TP1
gcuggucaaagggaaccacggcugucuuccuugaag......................	1	0	TP1
.....................................uuugguccccuuuaaccagc.	3	0	TP1
.....................................uuugguccccuuuaaccagcu	46	0	TP1
.....................................uuugguccccuuuaaccagcA	1	1	TP1
gcuggucaaagggaaccacg......................................	2	0	BS1
gcuggucaaagggaaccacggc....................................	37	0	BS1
gcuggucaaagggaaccacggcu...................................	27	0	BS1
gcuggucaaagggaaccacggcC...................................	1	1	BS1
gcuggucaaagggaaccacggcA...................................	1	1	BS1
gcuggucaaagggaaccacggcuU..................................	3	1	BS1
gcuggucaaagggaaccacggcugu.................................	1	0	BS1
gcuggucaaagggaaccacggcugucuuc.............................	1	0	BS1
gcuggucaaagggaaccacggcugucuuccuugaag......................	2	0	BS1
..uggucaaagggaaccacggc....................................	1	0	BS1
..................cggcugucuuccuugaagg.....................	1	0	BS1
....................................Cuuugguccccuuuaaccagcu	1	1	BS1
.....................................uuugguccccuuuaaccagc.	23	0	BS1
.....................................uuugguccccuuuaaccagcA	11	1	BS1
.....................................Cuugguccccuuuaaccagcu	1	1	BS1
.....................................uuCgguccccuuuaaccagcu	1	1	BS1
.....................................uuugguccccuuuaaccagcu	554	0	BS1
.....................................uuuggucUccuuuaaccagcu	1	1	BS1
......................................uugguccccuuuaaccagcu	2	0	BS1
....ugguccccuuuaaccagcu	4	0	BS1
gcuggucaaagggaaccacggc....................................	6	0	TP2
gcuggucaaagggaaccacggcu...................................	15	0	TP2
.....................................uuugguccccuuuaaccagcu	15	0	TP2
