# Supplementary material for: Differential expression of conserved and novel microRNAs during tail regeneration in the lizard Anolis carolinensis
Source: BMC Genomics. 2016 May 5;17:339. doi: 10.1186/s12864-016-2640-3 (PMC4858913; doi:10.1186/s12864-016-2640-3)
Supplement: Additional file 9: Figure S2. — Folding structure and read alignment for putative novel miRNA 5_10675.(PDF 202 kb) [file 12864_2016_2640_MOESM9_ESM.pdf]

miRBase precursor : 5_10675
Total read count : 1783
5_10675 read count : 1631
remaining reads : 152

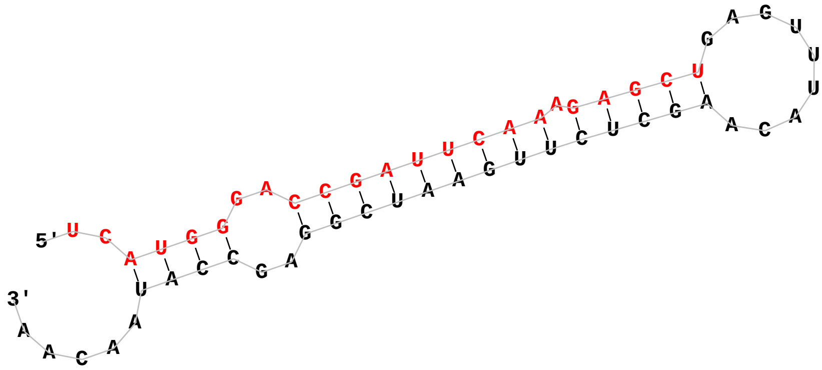

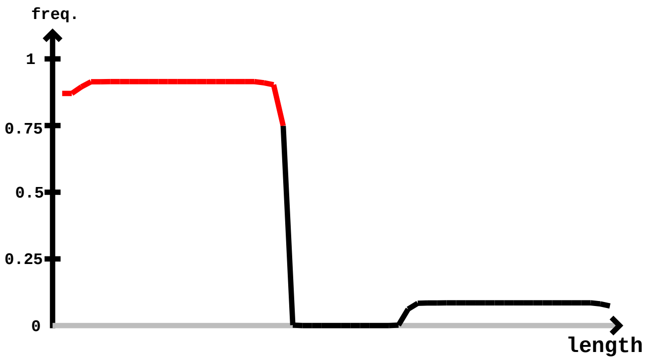

5_10675

5'- ucaugggaccgauucaaagagcugaguuuacaagcucuugaaucggagccauaacaa -3' exp

..((((..(((((((((.(((((.........))))))))))))))..)))).....	reads	mm	sample
ucaugggaccgauucCaagagc...................................	54	1	BS2
ucaugggaccgauucCaagagcu..................................	333	1	BS2
ucaugggaccgauucaaagagcu..................................	7	0	BS2
.caugggaccgauucCaagagc...................................	1	1	BS2
.caugggaccgauucaaagagcu..................................	2	0	BS2
.caugggaccgauucCaagagcu..................................	2	1	BS2
..augggaccgauucCaagagcu..................................	1	1	BS2
...................................Ccuugaaucggagccauaaca.	1	1	BS2
...................................ucuGgaaucggagccauaacaa	2	1	BS2
...................................ucuugaaucggagccauaacaC	1	1	BS2
...................................ucuugaaucggagccauaacaa	17	0	BS2
....................................cuugaaucggagccauaacaa	7	0	BS2
.....................................uugaaucggagccauaacaa	1	0	BS2
ucaugggaccgauucCaagagc...................................	12	1	TP3
ucaugggaccgauucaaagagcu..................................	4	0	TP3
ucaugggaccgauucCaagagcu..................................	39	1	TP3
ucaugggaccgauucaaagagcuA.................................	1	1	TP3
...................................ucuugaaucggagccauaacaa	1	0	TP3
ucaugggaccgauucCaagagc...................................	49	1	MUS
ucaugggaccgauucaaagagc...................................	1	0	MUS
ucaugggaccgauucaaagagcu..................................	1	0	MUS
ucaugggaccgauucCaagagcu..................................	106	1	MUS
.caugggaccgauucCaagagcu..................................	4	1	MUS
..augggaccgauucCaagagc...................................	3	1	MUS
..augggaccgauucCaagagcu..................................	3	1	MUS
...................................ucuugaaucggagccauaaca.	2	0	MUS
...................................ucuugaaucggagccauaacaa	5	0	MUS
....................................cuugaaucggagccauaacaa	3	0	MUS
ucaugggaccgauucaaaga.....................................	6	0	BRN
ucaugggacUgauucaaagag....................................	1	1	BRN
ucaugggaccgauucaaagag....................................	5	0	BRN
ucaugggaccgauucCaagag....................................	5	1	BRN

5_10675

```
ucaugggaccgauucaaagagcugaguuuacaagcucuugaaucggagccauaacaa
ucaugggaccgauucaaagagc...................................   20    0    BRN
ucaugggaccgauucCaagagc...................................   38    1    BRN
ucaugggaccgauucaaagagU...................................   2     1    BRN
ucaugggaccgauucaaagagA...................................   7     1    BRN
ucaugggaccgauucCaagagcu..................................   80    1    BRN
ucaugggaccgauucaaagagcu..................................   53    0    BRN
ucaugggaccgauucaaagagcuU.................................   1     1    BRN
.caugggaccgauucaaagagc...................................   1     0    BRN
.caugggaccgauucCaagagc...................................   1     1    BRN
.caugggaccgauucaaagagcA..................................   3     1    BRN
.caugggaccgauucaaagagcu..................................   13    0    BRN
.caugggaccgauucCaagagcu..................................   1     1    BRN
..augggaccgauucaaagU.....................................   1     1    BRN
..augggaccgauucCaagagc...................................   1     1    BRN
..augggaccgauucaaagagc...................................   1     0    BRN
..augggaccgauucaaagagcu..................................   4     0    BRN
..augggaccgauucCaagagcu..................................   2     1    BRN
...................................ucuugaaucggagccauaac..   3     0    BRN
...................................ucuGgaaucggagccauaac..   1     1    BRN
...................................ucuugaaucggagccauaacU.   1     1    BRN
...................................ucuGgaaucggagccauaaca.   1     1    BRN
...................................ucuGgaaucggagccauaacaa   19    1    BRN
...................................ucuugaaucggagccauaacaa   14    0    BRN
....................................cuugaaucggagccauaac..   2     0    BRN
....................................cuugaaucggagccauaaca.   3     0    BRN
....................................cuugaaucggagccauaacaa   12    0    BRN
....................................cuGgaaucggagccauaacaa   2     1    BRN
.....................................uugaaucggagccauaaca.   1     0    BRN
.......................................gaaucggagccauaacaa   1     0    BRN

ucaugggaccgauucCaagagc...................................   3     1    TP2
ucaugggaccgauucaaagagcu..................................   4     0    TP2
ucaugggaccgauucCaagagcu..................................   10    1    TP2
...................................ucuugaaucggagccauaacaa   1     0    TP2

ucaugggaccgauucaaagag....................................   1     0    TP1
ucaugggaccgauucCaagagc...................................   8     1    TP1
ucaugggaccgauucCaagagcu..................................   47    1    TP1
ucaugggaccgauucaaagagcu..................................   1     0    TP1
..augggaccgauucCaagagcu..................................   3     1    TP1
...................................ucuGgaaucggagccauaac..   1     1    TP1
...................................ucuugaaucggagccauaacaa   4     0    TP1

ucaugggaccgauucaaaga.....................................   1     0    BS1
ucaugggaccgauucCaagagc...................................   22    1    BS1
ucaugggaccgauucCaagagcu..................................   262   1    BS1
ucaugggaccgauucCaagagcug.................................   1     1    BS1
.caugggaccgauucCaagagc...................................   1     1    BS1
.caugggaccgauucaaagagc...................................   1     0    BS1
.caugggaccgauucCaagagcu..................................   7     1    BS1
..augggaccgauucCaagagc...................................   1     1    BS1
..augggaccgauucCaagagcu..................................   9     1    BS1
..................................cucuugaaucggagccauaaca.   1     0    BS1
..................................cucuugaaucggagccauaacaa   2     0    BS1
...................................ucuGgaaucggagccauaaca.   1     1    BS1
...................................ucuugaaucggagccauaaca.   3     0    BS1
...................................ucuugaaucggagccauaacaa   13    0    BS1
...................................ucuGgaaucggagccauaacaa   1     1    BS1
....................................cuugaaucggagccauaacaa   2     0    BS1

ucaugggaccgauucCaagag....................................   1     1    BS3
ucaugggaccgauucCaagagc...................................   46    1    BS3
ucaugggaccgauucCaagagcu..................................   313   1    BS3
ucaugggaccgauucaaagagcu..................................   6     0    BS3
ucaugggaccgauucGaagagcu..................................   1     1    BS3
.caugggaccgauucCaagagc...................................   2     1    BS3
.caugggaccgauucCaagagcu..................................   4     1    BS3
.caugggaccgauucaaagagcu..................................   1     0    BS3
..augggaccgauucaaagagcu..................................   1     0    BS3
..augggaccgauucCaagagcu..................................   4     1    BS3
```

5_10675

```
ucaugggaccgauucaaagagcugaguuuacaagcucuugaaucggagccauaacaa
....gggaccgauucCaagagcu..................................    1     1     BS3
...................................ucuugaaucggagccauaacaa    15    0     BS3
....................................cuugaaucggagccauaacaa    8     0     BS3
```
