# Supplementary material for: Differential expression of conserved and novel microRNAs during tail regeneration in the lizard Anolis carolinensis
Source: BMC Genomics. 2016 May 5;17:339. doi: 10.1186/s12864-016-2640-3 (PMC4858913; doi:10.1186/s12864-016-2640-3)
Supplement: Additional file 10: Figure S3. — Folding structure and read alignment for putative novel miRNA miR GL343237.1_6814. (PDF 218 kb) [file 12864_2016_2640_MOESM10_ESM.pdf]

miRBase precursor : GL343237.1_6814
Total read count : 32
GL343237.1_6814 read count : 27
remaining reads : 5

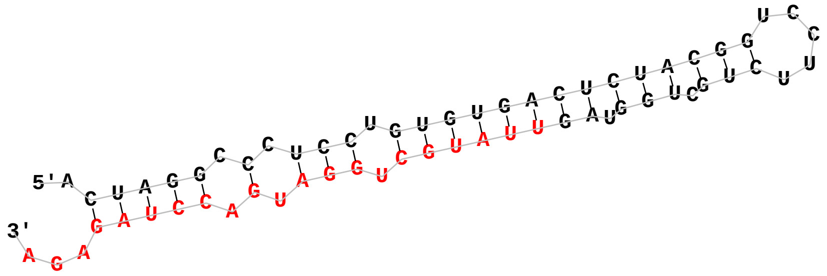

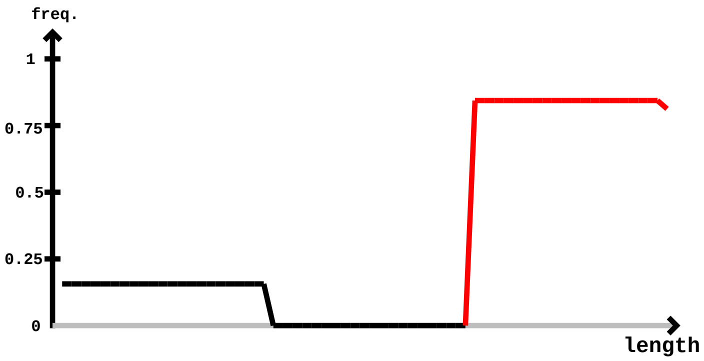

GL343237.1_6814

```
5'- acuaggcccuccugugugacucuacgguccuucugcugguaguuaugcuggaugaccuagaga   -3'    exp
    .(((((.(.(((.((((((((((((((.....))).))).)))))))).))).).)))))...     reads       mm          sample
    ..........................................uuaugcuggaugaccuagag.     1           0           BS2
    ..........................................uuaugcuggaugaccuagaga     6           0           BS2

    ..........................................uuaugcuggaugaccuagaga     1           0           BRN

    acuaggcccuccugugugacu..........................................     3           0           BS1
    ..........................................uuaugcuggaugaccuagaga     13          0           BS1

    acuaggcccuccugugugacu..........................................     2           0           BS3
    ..........................................uuaugcuggaugaccuagaga     6           0           BS3
```
